# Lacunar Infarcts, but Not Perivascular Spaces, Are Predictors of Cognitive Decline in Cerebral Small-Vessel Disease

**DOI:** 10.1161/STROKEAHA.117.017526

**Published:** 2018-01-31

**Authors:** Philip Benjamin, Sarah Trippier, Andrew J. Lawrence, Christian Lambert, Eva Zeestraten, Owen A. Williams, Bhavini Patel, Robin G. Morris, Thomas R. Barrick, Andrew D. MacKinnon, Hugh S. Markus

**Affiliations:** From the Department of Radiology, Imperial College NHS Trust, London, United Kingdom (P.B.); Atkinson Morley Regional Neuroscience Centre, St George’s University Hospitals NHS Foundation Trust, London, United Kingdom (S.T., A.D.M.); Neuroscience Research Centre, Institute of Molecular and Clinical Sciences, St George’s University of London, United Kingdom (C.L., E.Z., O.A.W., B.P., T.R.B.); Department of Psychology, King’s College Institute of Psychiatry, Psychology and Neuroscience, London, United Kingdom (A.J.L., R.G.M.); and Stroke Research Group, Clinical Neurosciences, University of Cambridge, United Kingdom (H.S.M).

**Keywords:** cerebral small vessel diseases, cognition, leukoaraiosis, magnetic resonance imaging, neuroimaging

## Abstract

**Background and Purpose—:**

Cerebral small-vessel disease is a major cause of cognitive impairment. Perivascular spaces (PvS) occur in small-vessel disease, but their relationship to cognitive impairment remains uncertain. One reason may be difficulty in distinguishing between lacunes and PvS. We determined the relationship between baseline PvS score and PvS volume with change in cognition over a 5-year follow-up. We compared this to the relationship between baseline lacune count and total lacune volume with cognition. In addition, we examined change in PvS volume over time.

**Methods—:**

Data from the prospective SCANS study (St Georges Cognition and Neuroimaging in Stroke) of patients with symptomatic lacunar stroke and confluent leukoaraiosis were used (n=121). Multimodal magnetic resonance imaging was performed annually for 3 years and neuropsychological testing annually for 5 years. Lacunes were manually identified and distinguished from PvS. PvS were rated using a validated visual rating scale, and PvS volumes calculated using T1-weighted images. Linear mixed-effect models were used to determine the impact of PvS and lacunes on cognition.

**Results—:**

Baseline PvS scores or volumes showed no association with cognitive indices. No change was detectable in PvS volumes over the 3 years. In contrast, baseline lacunes associated with all cognitive indices and predicted cognitive decline over the 5-year follow-up.

**Conclusions—:**

Although a feature of small-vessel disease, PvS are not a predictor of cognitive decline, in contrast to lacunes. This study highlights the importance of carefully differentiating between lacunes and PvS in studies investigating vascular cognitive impairment.

## Abstract 2

Supplemental Digital Content is available in the text.

Cerebral small-vessel disease (SVD) is a term used to describe a group of pathological processes that affect the perforating cerebral arterioles and capillaries. Many brain parenchymal pathologies can occur, including small infarcts, microbleeds, ischemic demyelination with axonal loss, and diffuse brain atrophy.^1^ Clinically SVD presents with lacunar strokes, which represent ≈20% of all ischemic strokes, and it is the major cause of vascular cognitive impairment.^2^

Enlarged perivascular spaces (PvS) visible on magnetic resonance imaging (MRI) are a feature of SVD and vascular dementia^2^ and are associated with lacunar stroke and T2 white matter hyperintensities (WMH).^3^ The significance of MRI-visible PvS in SVD, however, remains controversial.^2^ PvS are pial-lined interstitial fluid-filled cavities that surround penetrating arteries, arterioles, veins, and venules.^4^ Their relationship with cognitive impairment in SVD remains uncertain, and the few studies that have investigated this have yielded inconsistent results.^5–8^

This inconsistency may be because of the difficulty in distinguishing between lacunes and PvS. Lacunes are subcortical cavities which occur in the region of a previous acute small deep brain infarct or hemorrhage in the territory of a perforating arteriole^2^ and often look very similar to PvS. Lacunes are thought to impair cognition by disrupting white matter pathways.^9^ A failure to distinguish between them adequately may reduce the sensitivity of studies and clinical trials investigating cognitive impairment in SVD.

Another reason for the inconsistent association between PvS and cognition may be because most previous studies have used rating scales which are operator dependant and preferentially use T2-weighted MRI to assess PvS.^6,7,10^ PvS are difficult to rate in patients with moderate-to-severe SVD,^10^ as they are often obscured by WMH leading to an under or overestimation of PvS load. In addition, the rating scales currently used have a relatively narrow range (scores between 0 and 4) making longitudinal evaluation difficult. Measuring volumes using T1-weighted imaging is an alternative method to assess PvS load^11^ and may be more accurate in patients with SVD and WMH. In addition, it makes the longitudinal evaluation of PvS possible in a way that will be more sensitive to change than rating scales. To date, there are little data on whether PvS volumes change over time and whether they are associated with cognition.

The aim of this study is to further understand the clinical significance of PvS in symptomatic SVD. We carefully distinguished between lacunes and PvS and then investigated the relationship between PvS at baseline and cognitive change over a 5-year follow-up period in symptomatic SVD using both validated rating scales and computational measurements of PvS volumes. We compared this to the relationship between lacunes at baseline and cognitive change in the same population. In addition, we examined the change in PVS volume over time.

## Methods

The data that support the findings of this study are available from the corresponding author on reasonable request.

### Participants

Patients with SVD were recruited as part of the prospective SCANS study (St George’s Cognition and Neuroimaging in Stroke).^12^ Recruitment was from 3 hospitals covering a contiguous catchment area in South London (St George’s, King’s College, and St Thomas’ Hospitals). Inclusion criteria comprised a clinical lacunar stroke syndrome^2^ with an anatomically corresponding lacunar infarct on MRI in addition to confluent WMH on MRI (Fazekas grade ≥2).^13^ Exclusion criteria were any cause of stroke mechanism other than SVD (eg, cardioembolic source or extra- or intracerebral artery stenosis of >50%), other major central nervous system disorders, major psychiatric disorders, any other cause of white matter disease, contraindications to MRI, or nonfluent in English. The study was approved by the local ethics committee, and all patients gave written informed consent. MRI acquisitions and cognitive assessments were performed at least 3 months after the last stroke to exclude acute effects on cognition. All patients were also screened for cardiovascular risk factors including hypertension (defined as systolic blood pressure >140 mm Hg or diastolic >90 mm Hg or treatment with antihypertensive drugs), hypercholesterolemia (defined as a serum total cholesterol >5.2 mmol/L or treatment with a statin), diabetes mellitus, and smoking.

Participants were invited back annually for repeated cognitive testing and MRI scanning for a period of 3 years. Subsequently, 2 further annual assessments of cognitive function were conducted at years 4 and 5.

A total of 121 subjects were recruited. Of these, 103 attended >1 cognitive assessment. Eighteen subjects only attended 1 assessment because of death (n=7), formal study withdrawal (n=6), house move (n=1), lost to follow-up (n=2), and withdrawal from full neuropsychological testing (n=2). Of the 103 subjects who attended cognitive assessments more than once, MRI data at multiple time points were available for 99, 98 at year 1, 77 at year 2, and 71 at year 3. One subject attended the baseline and missed the year 1 follow-up, but attended all subsequent sessions. Four subjects missed the year 2 follow-up, but subsequently attended at year 3. Four subjects withdrew from imaging but remained in the study for neuropsychological testing.

### MRI Acquisition

Images were acquired on a 1.5-T scanner (General Electric, Milwaukee, WI). All image sequences were acquired across the whole brain, and total imaging time was ≈45 minutes. The imaging protocol included T2-weighted, fluid-attenuated inversion recovery, gradient echo, and 3-dimensional T1-weighted sequences. Further details on the imaging protocol are given in the online-only Data Supplement and Lawrence at al.^12^

### Image Processing

**Figure 1.**
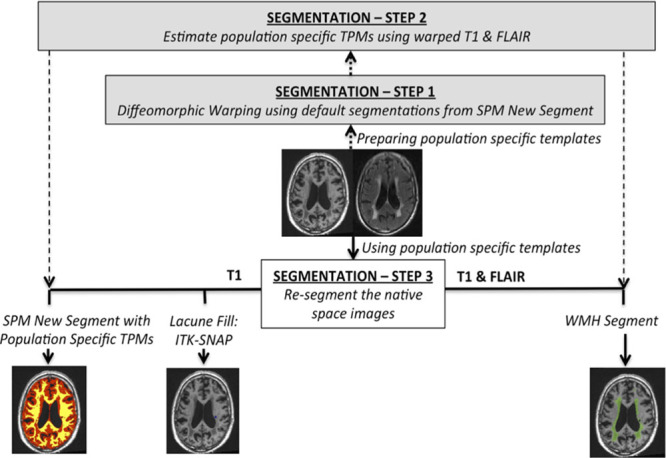
Summary of preprocessing pipeline. Please refer to Lambert et al^14^ for further details. FLAIR indicates fluid-attenuated inversion recovery; SPM, statistical parametric mapping; TPM, tissue probability map; and WMH, white matter hyperintensities.

Image processing was performed using the (SPM)8 software package (http://www.fil.ion.ucl.ac.uk/spm/software/spm8/). A summary is provided in Figure 1.^14^ Further details are given in the online-only Data Supplement.

### Identification of Lacunes and PvS

Lacunes were identified in native subject space by a consultant neuroradiologist (A.D.M.; blinded to the patient and cognitive data), using a multimodality view with T1-weighted, T2-weighted, and fluid-attenuated inversion recovery images. Lacunes were defined as subcortical, fluid-filled (similar signal as cerebrospinal fluid [CSF]) cavities (<15 mm in diameter) thought to be present in the region of a previous acute small deep brain infarct or hemorrhage in the territory of a perforating arteriole.^2^ Lacunes were distinguished from PvS using several criteria outlined in Table 1. A few cavities were excluded from the analysis if it was uncertain whether it was a PvS or a lacune.

**Table 1.**
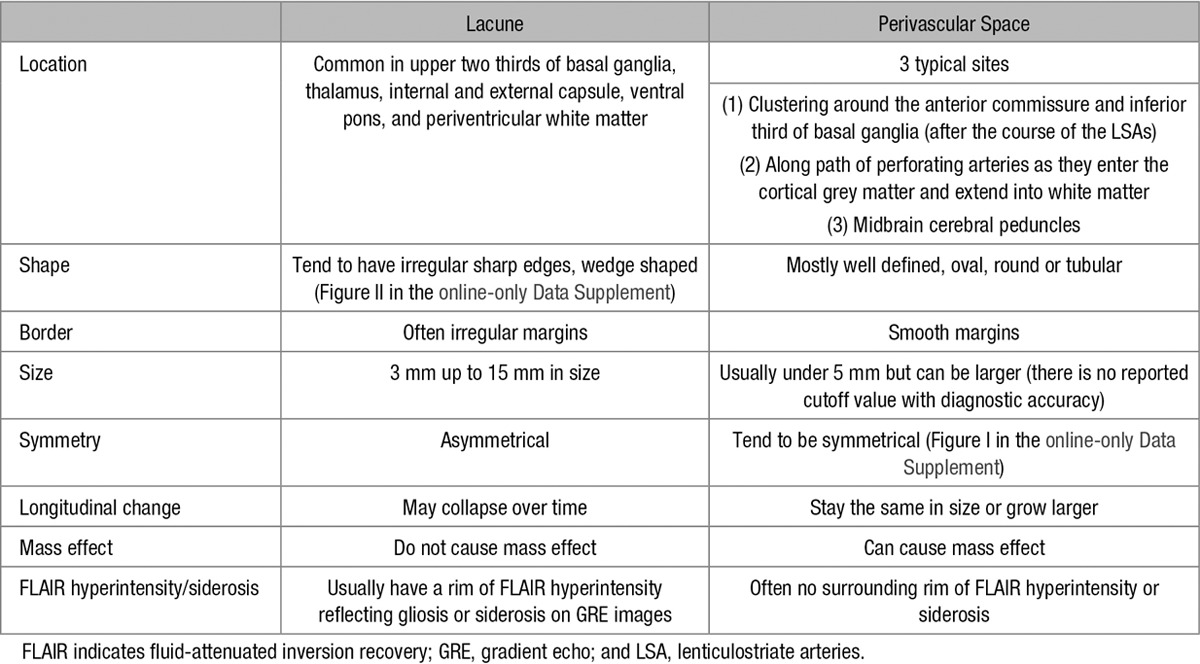
The Differences Between Lacunes and Perivascular Spaces on Conventional Clinical Imaging

### PvS Rating Scale

Images were rated by a trained observer using a validated visual rating scale^10^ using the recommended user guide (http://www.sbirc.ed.ac.uk/documents/epvs-rating-scale-user-guide.pdf).^10^ T2-weighted magnetic resonance scans (with T1-weighted and fluid-attenuated inversion recovery imaging also available) were used for analysis. Basal ganglia and centrum semiovale PvS were rated from 0 (none), 1 (1–10), 2 (11–20), 3 (21–40), and 4 (>40), by assessing and scoring each hemisphere separately and then using the hemisphere with the higher score. Midbrain PvS were rated 0 (none visible) or 1 (visible). PvS at the level of the anterior commissure were excluded from the overall rating. The scores from each region were added together to provide a total PvS score (EPVS) for each scan. Interrater reliability metrics were checked by 2 raters using 30 randomly selected scans. Both raters were blinded to the others ratings.

### PvS Volumes

Lacunes were first manually identified by an experienced neuroradiologist (A.D.M.) using criteria outlined above and manually delineated using ITK-SNAP (http://www.itksnap.org). The signal intensities of PvS spaces tend to be identical to or lower than those of CSF. Therefore, to create PvS maps, we used the already created CSF maps. The manually identified lacunes, the ventricles, and CSF surrounding the large vessels and outside the brain were removed from the CSF maps to create PvS maps. Each PvS map was manually inspected to ensure that only PvS were included. PvS volumes were calculated in individual subject space by summing these binarized corrected maps. Volumes were then normalized with respect to total brain volume. Inter- and intra-rater reliability metrics were checked by 2 raters using 20 randomly selected scans across all time points. Both raters were blinded with respect to subject and time point of each scan and results of cognitive testing.

### Neuropsychological Assessment

Cognitive assessment was performed annually using well-established standardized tests to include measures sensitive to the pattern of cognitive impairment associated with SVD.^12^ Tasks were grouped into broad cognitive functions, and task performance was age scaled using published normative data, transformed into *z* scores, and aggregated to construct the cognitive indices of executive function and processing speed by averaging across the component test measures for each subject, with a Global Function index as an amalgam of all measures. Further details on the individual tasks used and the cognitive assessment are given in the online-only Data Supplement.

### Statistical Analysis

Statistical analysis was performed in R version 3.1.2 (www.r-project.org).^15^ Because of the hierarchical nature of the data, we used linear mixed-effects modeling to estimate change over the follow-up period in our cognitive measures using the lme4 package (version 1.1–7) in R.^16^ Specifically, we used a random intercept and random slope model which permits the estimation of an average slope over 3 years for cognitive measures across the whole cohort while allowing for interindividual variability.^17^ The time of scan (in years from baseline) served as a within-subject variable. The neuropsychological scores (executive function, processing speed, and global function) were set as dependent variables separately. In all models, we controlled for age, sex, WMH volume, and number of cerebral microbleeds (CMBs). For each dependent variable, we first analyzed the main effects of baseline lacunes and PvS on baseline cognitive performance. In the same models, baseline MRI predictor×time interactions were explored to reveal the influence of baseline MRI markers on the rate of cognitive change. Our approach accommodates for patient dropout during follow-up with the assumption that unobserved measurements are missing at random.

Linear mixed-effect modeling (using a random intercept and random slope model) was also used to estimate change in PvS volume over a 3-year time period. To investigate the effect of change in PvS volume on cognition, PVS volumes were modeled as continuous time-varying variables, that is, decomposed into static (within-subject mean) and dynamic (residual between time-varying measurement and within-subject mean) components. This was done to assess the independent contribution of change in PVS volume on change in cognition. MRI predictor×time interactions were explored to reveal the influence of PvS volumes on the rate of cognitive change. Parameter estimates are summarized by their means and uncertainty, as expressed by the 95% confidence interval (95% CI).

For PvS rating scales, we calculated interobserver weighted κ for agreement using the psych package in R.^18^

Estimates were considered statistically significant when CIs excluded zero.

## Results

Demographics at baseline are given in Table 2. Patients who left the study (for any reason) had a significantly higher mean WMH load (*P*<0.004), a higher lacune load (*P*<0.013), a higher mean Rankin disability score (*P*<0.026), and a lower mean Mini-Mental Test Examination (*P*<0.004) score at baseline when compared with patients who attended all time points. There were, however, no significant differences in baseline brain volume or other demographic characteristics in patients that left the study

**Table 2.**
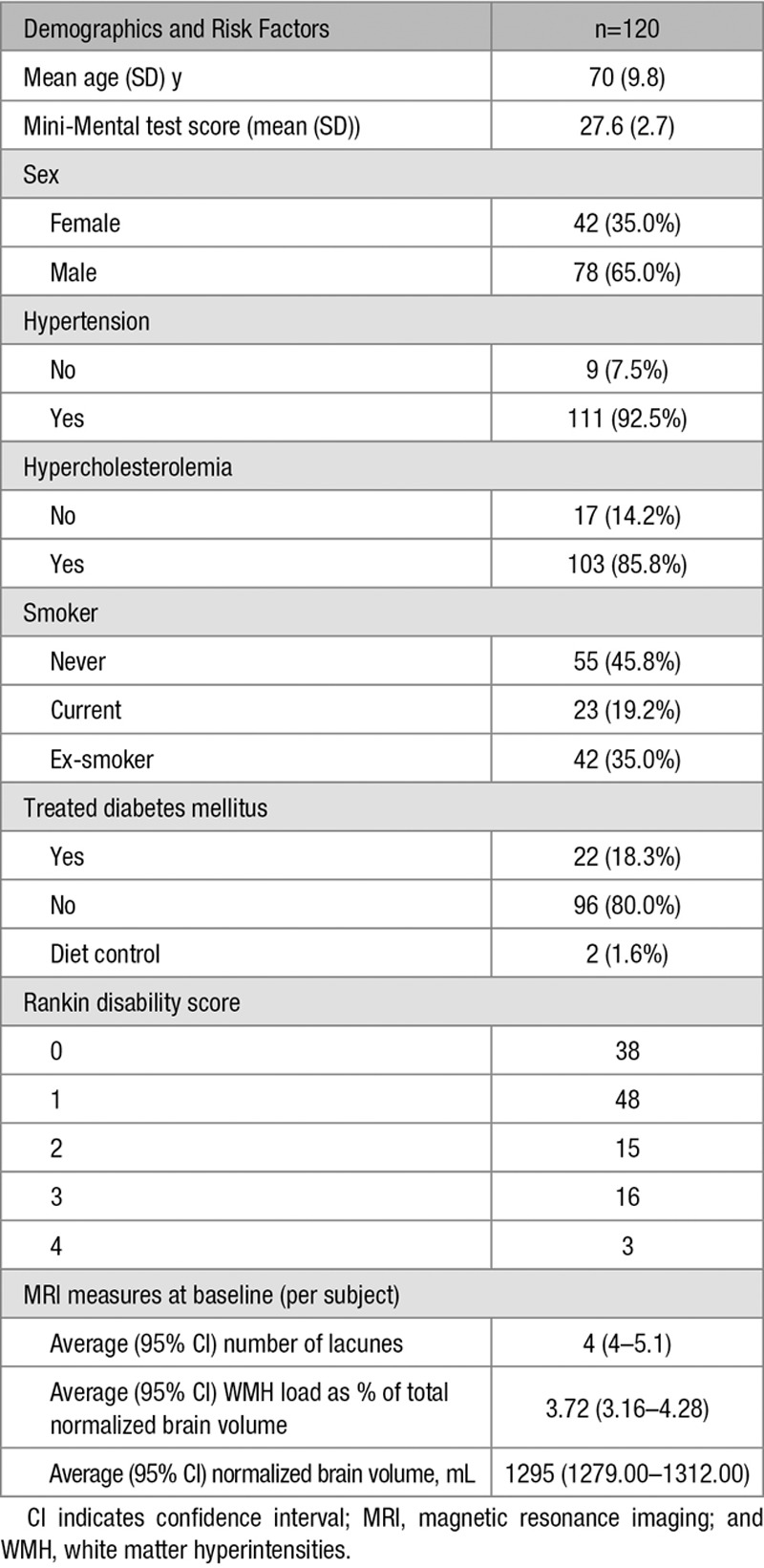
Patient Demographics at Baseline

### Perivascular Spaces

Of the 120 subjects, 1 had a total PvS score of 1, 7 had a score of 2, 34 had a score of 3, 23 had a score of 4, 39 had a score of 5, 12 had a score of 6, 3 had a score of 7, and 1 had a score of 8. Total PvS score at baseline was normally distributed with a mean (SD) of 4.2 (1.3). Baseline total PvS score was correlated with age (Spearman ρ=0.183; *P*=0.045), baseline lacunes (Spearman ρ=0.372; *P*<0.001), baseline CMBs (Spearman ρ=0.341; *P*<0.001), and baseline WMH volume (Spearman ρ=0.273; *P*=0.003). It was not associated with brain volume.

Mean total PvS volume was 160.6 mm^3^ (SD). Baseline total PvS volume was correlated with baseline lacunes (Pearson *r*=0.365; *P*<0.001) and baseline CMBs (Pearson *r*=0.189, *P*=0.038) but not with age, WMH volume, or brain volume.

There was a good correlation between total PvS scores and PvS volume (Spearman ρ=0.582; *P*<0.001). The estimated κ statistic measurements (confidence boundaries) for PvS rating scores were 0.40 (0.18–0.63) for centrum semiovale PvS and 0.33 (0.06–0.60) for basal ganglia PvS. The interrater reliability metrics for PvS volumes were SEM=2 mm^3^, mean variability=4.32% (SD=4.19%), and Intraclass correlation coefficient=0.99.

No significant change in total PVS volume over the 3-year observational period (for which MRI data were available) was demonstrated. The estimate of average (95% CI) annual change was −1.93 mm^3^ (−8.82 to 4.94). There was no evidence that change in PvS volume predicted a change in cognitive indices over the same follow-up period. The estimated effects (CI) of change in PvS volume on the slope of executive function, processing speed, and global function are 1.4×10^−3^ (−6.0×10^−5^ to 2.9×10^−3^), −2.3×10^−4^ (−1.4×10^−3^ to 9.8×10^−4^), and 6.0×10^−4^ (1.7×10^−4^ to 1.4×10^−3^), respectively.

### Lacunes

At baseline, cavitated lacunes were present in 99 (83%) of 120 subjects. Although all patients had clinical lacunar stroke syndrome (with corresponding MRI lacunar infarction), not all lesions detected on acute diffusion-weighted imaging subsequently cavitate on T1-weighted images.^19^ The mean (SD) number of lacunes was 4.18 (5.44). The mean (SD) total lacune volume as a percentage of total brain volume was 0.0754 (0.0979). The distributions of lacune count and volume were skewed and were therefore log_10_ transformed for statistical analysis. The number and distribution of lacunes at baseline has been described elsewhere.^9^

Over the 3-year imaging observational period, 74 new lacunes were observed in 27 patients, of which 66 were supratentorial and 8 were in the cerebellum or brain stem. A single new lacune was found in 10 subjects, 2 in 9 subjects and ≥3 (maximum 9) in 8 subjects.

### Change in Cognitive Measures

There was strong evidence of a decline in executive function, processing speed, and global function (*z* scores) over the course of the observational period. The average (95% CI) annual change for Executive Function was −4.2×10^−2^ (−4.2×10^−2^ to −0.4×10^−2^), for Processing Speed, −5.1×10^−2^ (−8.0×10^−2^ to −2.3×10^−2^), and for Global Function, −2.7×10^−2^ (−4.6×10^−2^ to −0.9×10^−2^).

### Associations Between PvS and Cognition

**Figure 2.**
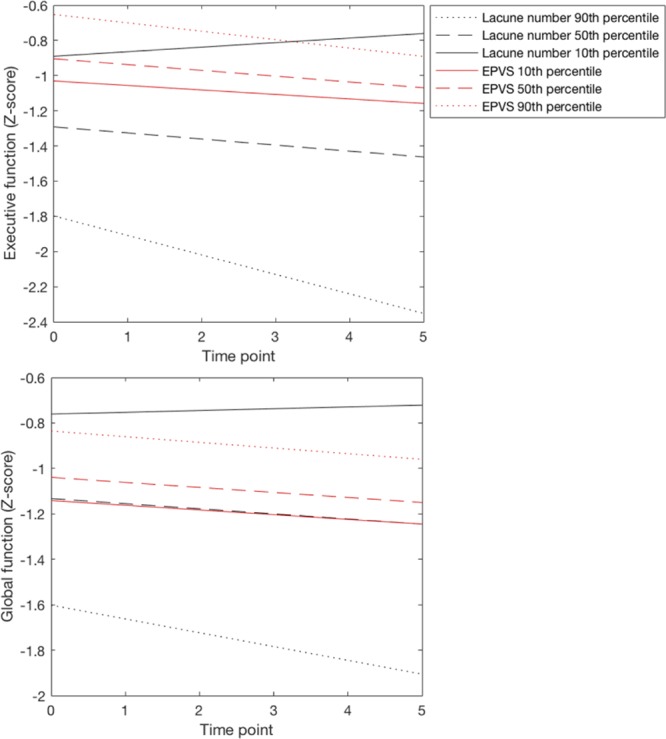
The estimated marginal effect of baseline lacune number and perivascular spaces score (EPVS) on Executive and global function over a 5-year follow-up period. We chose the 10th, 50^th^, and 90th percentiles of baseline lacunes (0, 2, 10) and EPVS (3, 4, 6) to display effects. Values for all other covariates in the model were set to their sample average.

**Figure 3.**
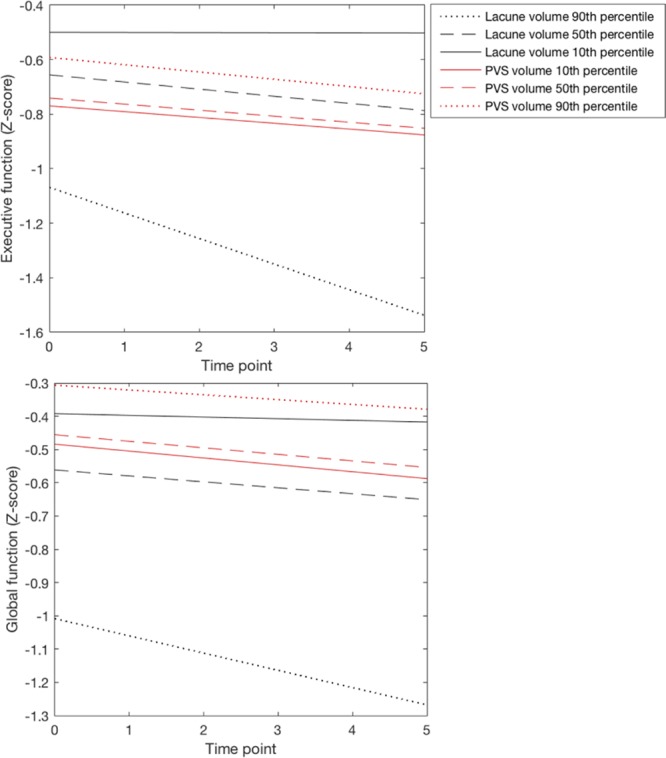
The estimated marginal effect of baseline lacune volume and perivascular spaces (PvS) volume on Executive and global function over a 5-year follow-up period. We chose the 10th, 50th, and 90th percentiles of baseline lacune volume (0, 0.47, 1.07) and PVS volume (20, 82, 406) to display effects. Values for all other covariates in the model were set to their sample average.

Total PvS score at baseline was not associated with cognition at baseline or with longitudinal change in cognition over a 5-year follow-up period. PvS in the basal ganglia, centrum semiovale, or midbrain were also not associated with cognition. PvS volume at baseline was not associated with cognition (Table I in the online-only Data Supplement). To help visualize the effects of baseline PvS on cognition, Figures 2 and 3 show the estimated marginal effect of baseline PvS score and PvS volume on cognition over a 5-year follow-up period.

### Associations Between Lacunes and Cognition

Lacune number and volume at baseline were associated with all cognitive indices at baseline and were strongest for executive function, processing speed, and global function (Table I in the online-only Data Supplement). These associations survived inclusion of baseline WMH volume, brain volume, and CMBs in the model.

The number of lacunes at baseline explained some of the variability of the slope of executive function with an estimated effect (CI) of −1.3×10^−1^ (−2.0×10^−1^ to −0.5×10^−1^) and global function with an estimated effect (CI) of −6.4×10^−2^ (−1.1×10^−1^ to −1.8×10^−1^) over time (Table I in the online-only Data Supplement). The volume of lacunes at baseline explained some of the variability in the slope of executive function with an estimated effect (CI) of −2.0×10^−1^ (−3.5×10^−1^ to −4.5×10^−2^; Table I in the online-only Data Supplement). The estimated effect of lacunes on the slope of executive function and global function remained significant after including WMH, CMBs, and brain volume in the model. Baseline, CMBs, WMH, and brain volume did not have a significant effect on the change of cognition over time. The model was not significantly affected by collinearity (variance inflation factors were all under 2). To help visualize the effects of baseline lacunes on cognition, Figures 2 and 3 show the estimated marginal effect of baseline lacune number and lacune volume volume on cognition over a 5-year follow-up period.

## Discussion

We compared the impact of baseline PvS and lacunes in a population of patients with symptomatic SVD. We found that baseline lacunes had a significant effect on cognition, both at baseline and longitudinally, whereas PvS had no impact on cognition in the same cohort. PvS, however, do correlate with other MRI markers of SVD particularly lacunes and CMBs. This suggests that while PvS may be a marker of SVD severity, they are not associated with cognitive impairment.^9^

A few studies,^7,8^ however, have showed a positive association between PvS and cognitive impairment. Apart from confounding factors, a reason for this inconsistency may be because of the difficulty in differentiating between lacunes and PvS which may result in lacunes being included as PvS. In addition, the rating scales used in previous studies preferentially use T2-weighted images^3^ for analysis, which can often overestimate the burden of PvS in patients with severe white matter disease. Previous studies have suggested that the presence of lacunes and WMH was the main reason for discrepancy between observers.^10^ All our patients have moderate-to-severe SVD which may explain the only moderate reproducibility demonstrated for the visual rating scales. For this reason, in our study, we carefully differentiated between lacunes and PVS (Table 1) and used 2 different methods of quantifying PvS (a visual rating score and PvS volumes). Although the only moderate reproducibility may have reduced the ability to detect associations on visual rating, it would not affect the semiautomated volumetric measures.

Incident lacunes have been proposed as a disease biomarker in cerebral SVD.^20^ This study highlights the importance of carefully differentiating between lacunes and PvS, particularly in studies investigating vascular cognitive impairment. Failing to differentiate these MRI features will reduce the sensitivity to detect significant effects.

Our results did not demonstrate a significant change in PVS volume over a 3-year observational period. This may be because PvS have a slow growth rate and a longer period of imaging follow-up may be necessary to determine change in PvS volumes. Detailed 3-dimensional tracing of PvS is now possible using high-field strength MRI^21,22^ which will no doubt enable us to better study PvS thereby improving our understanding of their pathophysiological significance in SVD and other neurodegenerative diseases.

A limitation of previous studies of PvS is that rating scales may not be sensitive enough to evaluate the true burden of PvS. For this reason, we also used a volumetric measure of PvS which also allowed us to assess for longitudinal change. We however only used T1-weighted images for our analysis of PvS volumes as these images had the highest resolution in our data. In future studies, the data might be improved by acquiring other sequences including T2-weighted imaging with isotropic voxel dimensions, at higher resolutions.

A limitation of this study was that we had a relatively high dropout rate, although this is consistent with previous longitudinal studies in aging.^23^ Our analysis, using linear mixed-effect models provides inferences under the missing at random assumption. Although the majority of longitudinal studies in neuroimaging including clinical trials make this assumption,^24^ the possibility of data missing not at random is difficult to rule out. For example, patients who did not complete follow-up tended to be older and more disabled. This may have led to an underestimation of the rate of change in MRI markers and cognition. The handling of missing data continues to be a subject of discussion and alternative methodologies, for example, multiple imputation models may provide a more flexible approach. Future studies with larger sample sizes may be able to detect a small effect that may have been missed on this study.

We also assume linearity of change over time. In our data with a relatively short follow-up period, cognitive change is more parsimoniously described by a linear fit rather than a quadratic fit (judged by the small sample corrected Akaike Information Criterion).^25^ However, it is possible that with fewer dropouts or a longer period of follow-up period, or a more homogeneous disease stage among participants, nonlinearities may be more apparent and a quadratic or cubic fit may prove more appropriate. It should be noted that the processing speed index is made of aggregate scores from the following tasks: Wechsler Adult Intelligence Scale-III (Wechsler,^26^ 1997) Digit symbol substitution, Speed of Information Processing Task,^25^ and Grooved Pegboard Task,^27^ a combination which has good internal reliability.^12^ All of these tests have motor responses, in particular the Grooved Pegboard task which has a greater motor component than the other tests. A processing speed deficit may therefore include a component of motor slowing in SVD.

We tried to exclude patients with cerebral amyloid angiopathy by excluding patients with cortical hemorrhages or in whom the pattern of CMBs was in a lobar distribution, but it is possible that some patients did have coexistent cerebral amyloid angiopathy pathology as this becomes increasingly frequent with increasing age. To study a homogenous group of patients, we recruited only patients with symptomatic lacunar infarction confirmed on MRI and confluent leukoaraiosis. We recognize that this may limit the generalizability of the findings. However, the findings remain relevant to a large number of stroke patients—about 20% to 25% of all ischemic stroke is lacunar because of SVD and of these cases about half fall into the category of lacunar stroke and WMH.

## Conclusions

In conclusion, PvS, although a feature of SVD, are not associated with cognitive decline over a 5-year follow-up period. In contrast, lacunes are an important predictor of future cognitive decline. This study underlines the importance of carefully differentiating between lacunes and PvS in studies investigating vascular cognitive impairment.

## Sources of Funding

The SCANS (St Georges Cognition and Neuroimaging in Stroke) research study was supported by a Wellcome Trust grant (081589). Recruitment was supported by the English National Institute of Health Research (NIHR) Clinical Stroke Research Network. H.S. Mar research is supported by an NIHR Senior Investigator award and the Cambridge University Hospitals NIHR Comprehensive Biomedical Research Centre.

## Disclosures

None.

## Supplementary Material
