# Supplementary material for: Lacunar Infarcts, but Not Perivascular Spaces, Are Predictors of Cognitive Decline in Cerebral Small-Vessel Disease
Source: Stroke. 2018 Jan 31;49(3):586–93. doi: 10.1161/STROKEAHA.117.017526 (PMC5832012; doi:10.1161/STROKEAHA.117.017526)
Supplement: Supplementary file 1 [file str-49-586-s001.pdf]

SUPPLEMENTAL MATERIAL

Magnetic resonance imaging acquisition

Images were acquired on a 1.5T Signa HDxt scanner (General Electric, Milwaukee, WI, USA) with maximum gradient amplitude of $33mTm^{-1}$ and a proprietary head coil. All image sequences were acquired across the whole brain and total imaging time was approximately 45 minutes. The imaging protocol included: (1) Fluid Attenuated Inversion Recovery (FLAIR) sequence - TR/TE/TI=9000/130/2200ms, field-of-view (FOV) = $240x240mm^2$, matrix = 256x192, 28 axial slices of 5mm thickness. (2) Spoiled gradient echo recalled T1-weighted (SPGR) 3D coronal sequence - TR/TE=11.5/5ms, FOV=$240x240mm^2$, matrix=256x192, flip angle=18°, 176 coronal slices of 1.1mm thickness reconstructed to an in plane resolution of 1.1mm.

Image processing

The raw DICOMS were imported using the SPM8 software package (http://www.fil.ion.ucl.ac.uk/spm/software/spm8/). T1-weighted and FLAIR images were co-registered together using an affine transformation in SPM for each individual, before affine transformation to the same orientation as the MNI template and resliced to 1 mm isotropic resolution using 4th degree b-spline interpolation. The conventional SPM segmentation pipelines were adapted and optimised to our study population. This better captured population specific features (such as larger ventricles). To achieve this, a group average template was generated and all the T1-weighted and FLAIR images are warped to this space. The T1-weighted and FLAIR images in the group average space were then used to create population specific tissue probability maps (TPMs). The default SPM TPMs were replaced by the population specific TPMs[1] in the SPM segmentation algorithm to re-segment native space images to generate grey matter (GM), white matter (WM), cerebrospinal fluid (CSF) and WMH tissue class segmentation images. These were then combined with manually defined lacune ROIs to provide five tissue class images per individual (Figure 1 in main manuscript). The WMH segmentation maps were binarised at a threshold set for each individual by checking results manually to ensure accurate correspondence with lesions on the FLAIR image. Results were manually refined where necessary to optimise accuracy. For further details on image processing please refer to Lambert et al[1].

WMH volume

WMH masks were created using the above technique from the FLAIR images. The volumes at each time-point were calculated in native subject space by summing the binarised corrected segmentations. To adjust for the effects of head size and brain atrophy on WMH volume, we analysed WMH load by expressing WMH volume as a percentage of the volume of total white matter.

Estimation of brain volume

Brain volume at baseline was calculated using a fully automated program, SIENAX (Cross-sectional Structural Image Evaluation using Normalisation of Atrophy www.fmrib.ox.ac.uk/fsl)[2] on T1-weighted images.

Cerebral Microbleeds

Baseline CMBs were identified by a consultant neuroradiologist. The Brain Observer Microbleed Rating Scale (BOMBS) was used to identify and describe CMB locations. CMBs were defined as homogeneous round focal areas < 10 mm in diameter of low signal intensity on T2*-weighted GRE images. Only CMBs meeting the BOMBS "certain" criteria were analysed. Bilateral basal calcification, flow voids from blood vessels, and low signals averaging from adjacent bone were regarded as "uncertain" CMBs.

Neuropsychological assessment

Cognitive assessment was carried out annually using well established standardized tests to include measures sensitive to the pattern of cognitive impairment associated with SVD. Premorbid IQ was estimated using the National Adult Reading Test-Restandardised (NART-R)[3]. Tasks were grouped into broad cognitive functions as follows:

Executive function: Trail Making test, (Reitan 1996) Modified Wisconsin Card Sorting Test[4] and Phonemic Fluency (FAS) [5].

Processing speed: Wechsler Adult Intelligence Scale-III (Wechsler, 1997a) Digit symbol substitution [6], Speed of Information Processing Task [7] and Grooved Pegboard Task [8].

Task performance was age scaled using published normative data, transformed into z-scores and aggregated to construct the cognitive indices of executive function and processing speed by averaging across the component test measures for each subject, with a Global Function index as an amalgam of all measures. For further details on the cognitive assessment please refer to Lawrence et al [9].

Supplementary Table I: Estimates (confidence intervals) of the intercept and slope of cognitive indices over 5-year follow-up followed by the estimated effects (confidence intervals) of baseline lacunes and perivascular spaces on these values. Estimates with a significant effect are shown in bold. Lacunes number and volume had a significant effect on all cognitive indices at baseline. Lacune number explained some variability in the slope of executive function and global function in the model. PvS did not have a significant effect on the intercept or slope. Results were considered statistically significant when confidence intervals excluded zero.

	Executive Function	Processing Speed	Global Function
**Intercept**	**$-9.03x10^{-1}(-1.09x10^{0}, -7.12x10^{-1})$**	**$-9.64x10^{-1}(-1.12x10^{0}, -8.07x10^{-1)}$**	**$-6.51x10^{-1}(-4.56x10^{-1}, -1.20x10^{-2})$**
**Total PVS score**	$4.62x10^{-2}(-0192x10^{-2}, 9.95x10^{-2})$	$-8.91x10^{-2}(-2.08x10^{-1}, 2.96x10^{-2})$	$3.52x10^{-1}(-1.50x10^{-1}, 7.91x10^{-2})$
**Total PVS volume**	$5.80x10^{-4}$ $(-1.00x10^{-3}, 1.00x10^{-3})$	$2.01x10^{-4}(-1.10x10^{-3}, 6.01x10^{-4})$	$4.60x10^{-4}(-8.60x10^{-4}, 7.01x10^{-4})$
**Lacune number**	**$-8.69x10^{-1}(-1.35x10^{0}, -3.87x10^{-1})$**	**$-8.43x10^{-1}(-1.23x10^{0}, -4.58x10^{-1})$**	**$-6.94x10^{-1}(-1.07x10^{0}, -3.16x10^{-1})$**
**Lacune volume**	**$-1.22x10^{0}(-2.28x10^{0}, -2.07x10^{-1})$**	**$-1.45x10^{0}(-2.28x10^{0}, -6.23x10^{-1})$**	**$-1.15x10^{0}(-1.96x10^{0}, -3.40x10^{-1})$**
**Time (slope)**	**$-4x77x10^{-2}(-7.70x10^{-2}, 1.83x10^{-2})$**	**$-5.18x10^{-2}(-7.87x10^{-2}, -2.50x10^{-2})$**	**$-2.92x10^{-2}(-4.65x10^{-2}, -1.19x10^{-2})$**
**Total PVS score×Time**	$-7.21x10^{-3}(-2.95x10^{-2}, 1.51x10^{-2})$	$-5.03x10^{-3}(-1.56x10^{-2}, 2.56x10^{-2})$	$-1.44x10^{-3}$(-1.43x10-2, $1.15x10^{-2})$
**Total PVS volume×Time**	$-1.61x10^{-5}$(-1.69x10-^{4}, $1.47x10^{-4})$	$1.72x10^{-5}(-1.59x10^{-4}, 1.24x10^{-4})$	$-1.7x10^{-5}(-1.06x10^{-4}, 7.1x10^{-5})$
**Lacune number×Time**	**$-1.27x10^{-1}(-2.04x10^{-1}, -5.02x10^{-2})$**	$-1.88x10^{-2}(-9x53x10^{-2}, 5x78x10^{-2})$	**$-6.43x10^{-2}(-1.11x10^{-1}, -1x76x10^{-2})$**

**Lacune volume×Time**	**$-1.98x10^{-1}$($-3.51x10^{-1}$, -4x48x10^{-2})**	$-3.73x10^{-2}$($-1.87x10^{-1}$, $1.12x10^{-1}$)	$-6.70x10^{-2}$($-1.56x10^{-1}$, $2.18x10^{-2}$)

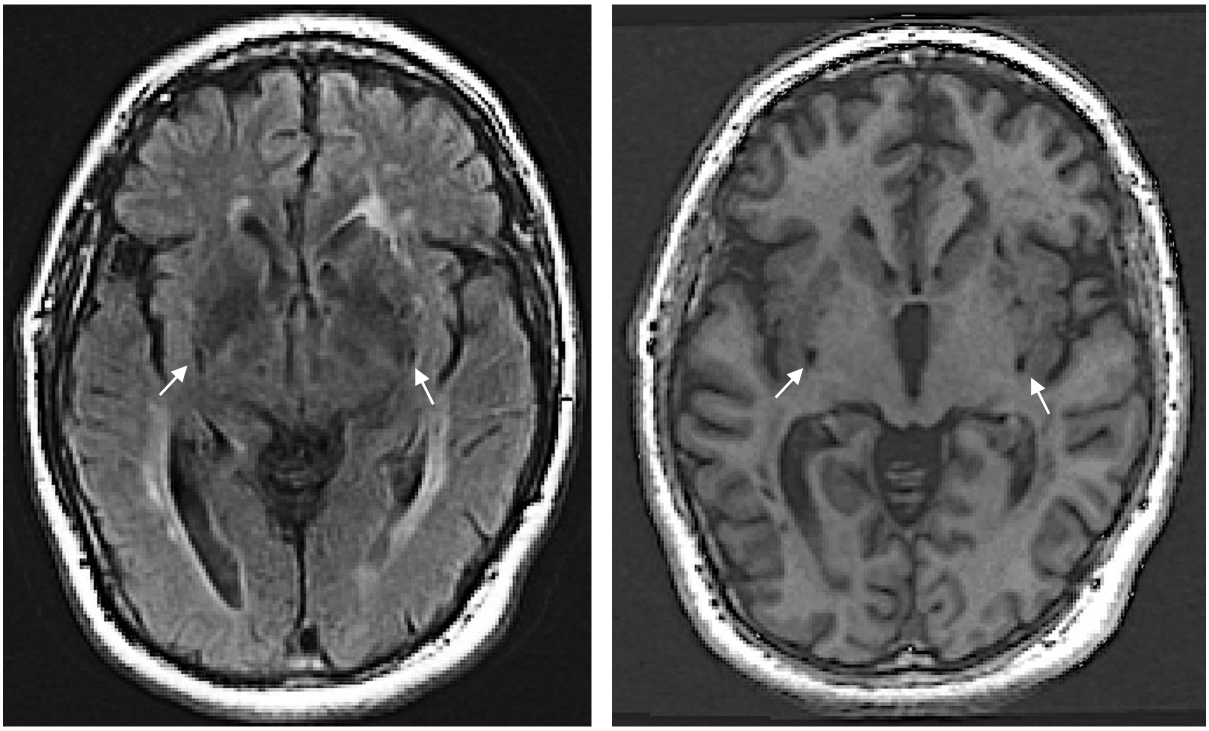

Supplementary Figure I: Axial FLAIR and T1-weighted images showing symmetrical perivascular spaces (arrows)

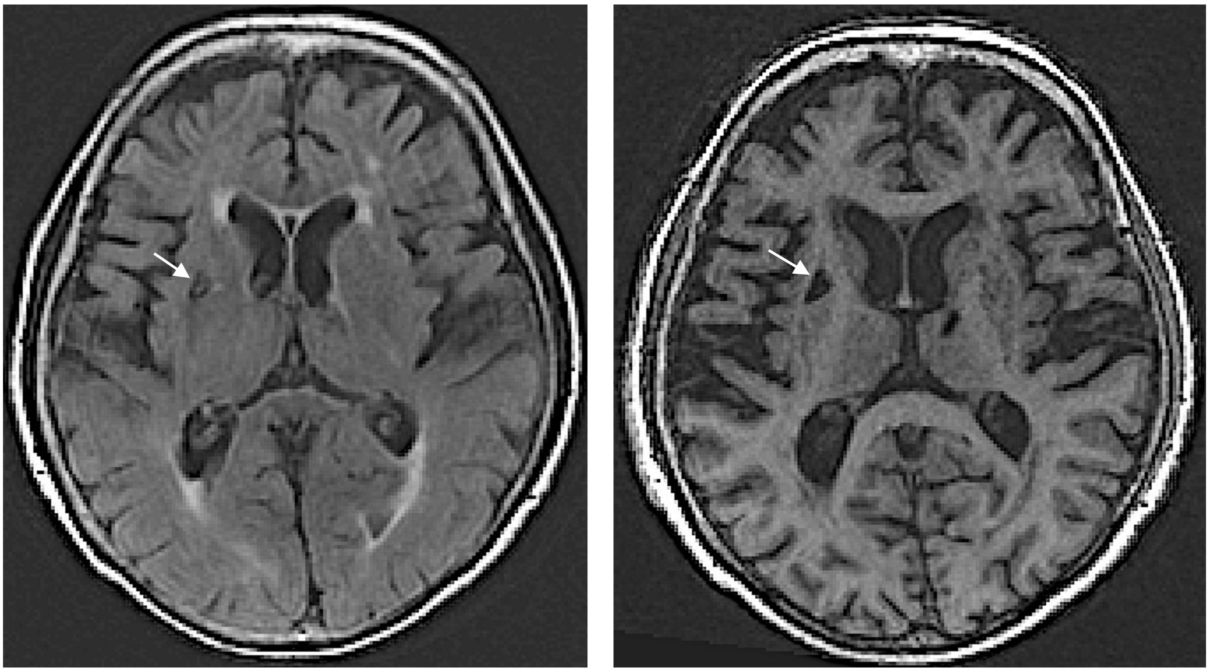

Supplementary Figure II: Axial FLAIR and T1-weighted images showing a wedge shaped lacune (arrows)

References:

1. Lambert C, Benjamin P, Zeestraten E, Lawrence AJ, Barrick TR, Markus HS. Longitudinal patterns of leukoaraiosis and brain atrophy in symptomatic small vessel disease. *Brain J. Neurol.* 2016;139:1136–1151.

2. Smith SM, Zhang Y, Jenkinson M, Chen J, Matthews PM, Federico A, et al. Accurate, robust, and automated longitudinal and cross-sectional brain change analysis. *NeuroImage*. 2002;17:479–489.

3. Nessol H, Willison JR. National Adult Reading Test(NART): Test Manual. 2nd Ed. Windsor, UK: NFER-Nelson; 1991.

4. Nagahama Y, Okina T, Suzuki N, Matsuzaki S, Yamauchi H, Nabatame H, et al. Factor Structure of a Modified Version of the Wisconsin Card Sorting Test: An Analysis of Executive Deficit in Alzheimer’s Disease and Mild Cognitive Impairment. *Dement. Geriatr. Cogn. Disord.* 2003;16:103–112.

5. Delis DC, Kaplan E, Kramer JH. Delis-Kaplan Executive Function System. San Antonio TX: Psychological Corporation; 2001.

6. Wechsler D. Wechsler Memory Scale WMS-III. 3rd Ed. San Antonio TX: Psychological Corporation; 1997.

7. Coughlan A, Oddy M, Crawford J. The BIRT Memory and information processing battery (B-MIPB). Wakefield. UK: The Brain Injury Rehabilitation Trust (BIRT); 2009.

8. Matthews C, Klove H. Instruction manual for the adult Neuropsychological test battery. Madison, WI: University of Wisconsin Medical School; 1964.

9. Lawrence AJ, Patel B, Morris RG, MacKinnon AD, Rich PM, Barrick TR, et al. Mechanisms of Cognitive Impairment in Cerebral Small Vessel Disease: Multimodal MRI Results from the St George's Cognition and Neuroimaging in Stroke (SCANS) Study. *PLoS ONE*. 2013;8:e61014.
